# Modeling undernutrition with enteropathy in mice

**DOI:** 10.1038/s41598-020-72705-0

**Published:** 2020-09-24

**Authors:** Emmeline Salameh, Marine Jarbeau, Fanny B. Morel, Mamane Zeilani, Moutaz Aziz, Pierre Déchelotte, Rachel Marion-Letellier

**Affiliations:** 1 Normandie Univ, INSERM Unit 1073, University of Rouen, 22 Boulevard Gambetta, 76000 Rouen, France; 2 Institute for Research and Innovation in Biomedicine (IRIB), University of Rouen, Rouen, France; 3 Nutrition Department, Nutriset S.A.S, Malaunay, France; 4 Anatomopathology, Rouen University Hospital, Rouen, France; 5 Nutrition Unit, Rouen University Hospital, Rouen, France

**Keywords:** Malnutrition, Gastrointestinal models

## Abstract

Undernutrition is a global health issue leading to 1 out 5 all deaths in children under 5 years. Undernutrition is often associated with environmental enteric dysfunction (EED), a syndrome associated with increased intestinal permeability and gut inflammation. We aimed to develop a novel murine model of undernutrition with these EED features. Post-weaning mice were fed with low-protein diet (LP) alone or combined with a gastrointestinal insult trigger (indomethacin or liposaccharides). Growth, intestinal permeability and inflammation were assessed. LP diet induced stunting and wasting in post-weaning mice but did not impact gut barrier. We therefore combined LP diet with a single administration of indomethacin or liposaccharides (LPS). Indomethacin increased fecal calprotectin production while LPS did not. To amplify indomethacin effects, we investigated its repeated administration in addition to LP diet and mice exhibited stunting and wasting with intestinal hyperpermeability and gut inflammation. The combination of 3-weeks LP diet with repeated oral indomethacin administration induced wasting, stunting and gut barrier dysfunction as observed in undernourished children with EED. As noninvasive methods for investigating gut function in undernourished children are scarce, the present pre-clinical model provides an affordable tool to attempt to elucidate pathophysiological processes involved in EED and to identify novel therapeutic strategies.

## Introduction

According to UNICEF, undernutrition is a major public health issue in low-income countries^1^. Inadequate dietary intake may lead to child stunting and/or wasting and is associated with an increased risk of impaired cognitive development, future chronic diseases, morbidity and mortality^1–3^. Undernutrition is often associated with environmental enteric dysfunction (EED) and both contribute to a vicious cycle that perpetuates stunting and induces cognitive shortfalls. EED is characterized by gut barrier dysfunction reflected, among other features, by intestinal hyperpermeability, gut inflammation and villus blunting^4,5^.

Murine models have been developed to dissect the impact of depleted diets on post-weaned mice^6–8^. Two inadequate diets common to children from low-resource countries are calories restricted (CR) and low protein (LP) diets. Mice exposed to both depletions exhibit weight loss and growth faltering^9–12^. These models share features of undernutrition related to anthropometric measures observed in humans but their effects on gut barrier function are more debated^6,9,10^. To impact gut barrier function on murine models, depleted diets has been combined with microbial infections to reflect environmental exposures experienced by malnourished children. Microbial challenges inducing gut inflammation include bacterial cocktail^9^ or parasites such as C*ryptosporidium parvum* or *Giardia*^13,14^. These murine models are relevant to EED but their reproducibility may be difficult because of differential microbial proliferation. In addition, inflammation induced by pathogens can induce more drastic inflammatory phenotypes compared to subclinical features observed in human EED.

We thus aimed to develop a novel murine model of undernutrition exacerbating EED features by dissecting the respective impact of undernutrition and enteric insults on gut barrier function. Undernutrition was induced by CR or LP diet. Small intestinal injury was induced by bacterial lipopolysaccharides (LPS) or indomethacin. We chose LPS because serum anti-LPS immunoglobulins in children are elevated and have been associated with poor growth outcomes^15–17^. Moreover, LPS is also well known to induce gut inflammation via Toll Like Receptor activation in vivo and in vitro^18^. Indomethacin, a non-steroidal anti-inflammatory drug, is commonly used to induce enteropathy in normo-nourished mice^19,20^. The aim of our study was to establish a model of undernutrition with EED features, which represents a crucial tool for research applications and further therapeutic approaches.

## Results

### Effect of a 3-weeks low protein diet and caloric restriction on growth and barrier function (Fig. 1)

**Figure 1:**
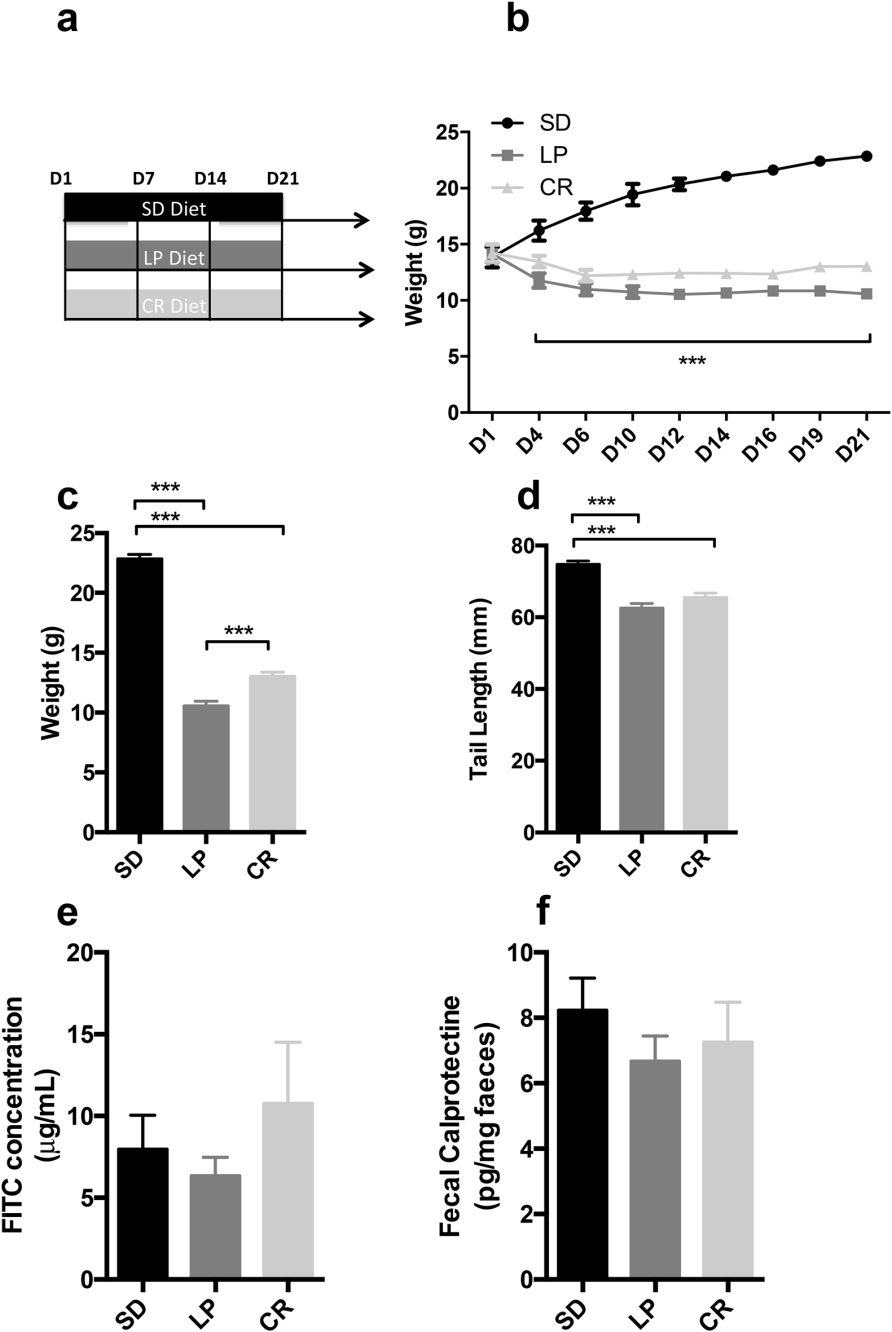
Growth, intestinal inflammation and permeability assessment in C57BL/6 mice fed a low protein, calorie restrictive or isocaloric standard diet. (**a**) 3-weeks-old mice were fed with standard, isocaloric low protein or 50% calorie restrictive diet post weaning for 21 days. (**b**) Body weight was recorded every 2 days until the end of the experimentation (n = 20 per group). Plots represent the mean ± SEM (***P < 0.001; 2-Way ANOVA). At D21, (**c**) body weight (n = 20 per group) and (**d**) tail length (n = 20 per group) were measured at the end of the experiment. Bars indicate the mean ± SEM One-way analysis of variance with post hoc Tukey’s test was performed (***P < 0.0001). (**e**) Bars indicate the mean ± SEM of the jejunal concentration of FITC into musculous side after 3 h after the beginning of ex vivo measurement of jejunal permeability using Ussing chamber at D21 (n = 20 per group). Kruskal Wallis test with post hoc Dunn's Multiple Comparison Test was performed. (**f**) Bars indicate the concentration of fecal calprotectin in mice at D21 (n = 20 per group). One-way analysis of variance with post hoc Tukey’s test was used. SD, Standard diet; LP, Low protein diet; CR, Caloric Restriction.

We first characterized the impact of 50% CR or LP diet on growth and gut barrier function in post-weaning mice compared to mice fed with SD (Fig. 1a). LP and CR groups had a lower weight compared to SD group (− 53% for LP, − 43% for CR, P < 0.0001 for both vs. CT—Fig. 1b,c). Mice fed with the LP diet had a lower weight over time compared to the calorie restricted group (10.6 g ± 0.4 for LP, 13.0 g ± 0.4, P < 0.0001—Fig. 1c). Both groups had also a significantly shorter tail length (− 16.3% for LP, − 12.4% for CR, P < 0.0001 for both vs. *SD*—Fig. 1d). No impact on jejunal permeability was observed (− 20.5% for LP, + 35.2% for CR, P = 0.8 vs. SD for both—Fig. 1e). No significant changes in fecal calprotectin were found among groups (− 18.9% for LP, − 11.9% for CR, P > 0.05 vs. SD for both—Fig. 1f).

### Effect of a 2-weeks low protein diet combined with lipopolysaccharide or indomethacin intraperitoneal single injection on growth and barrier function (Fig. 2)

**Figure 2:**
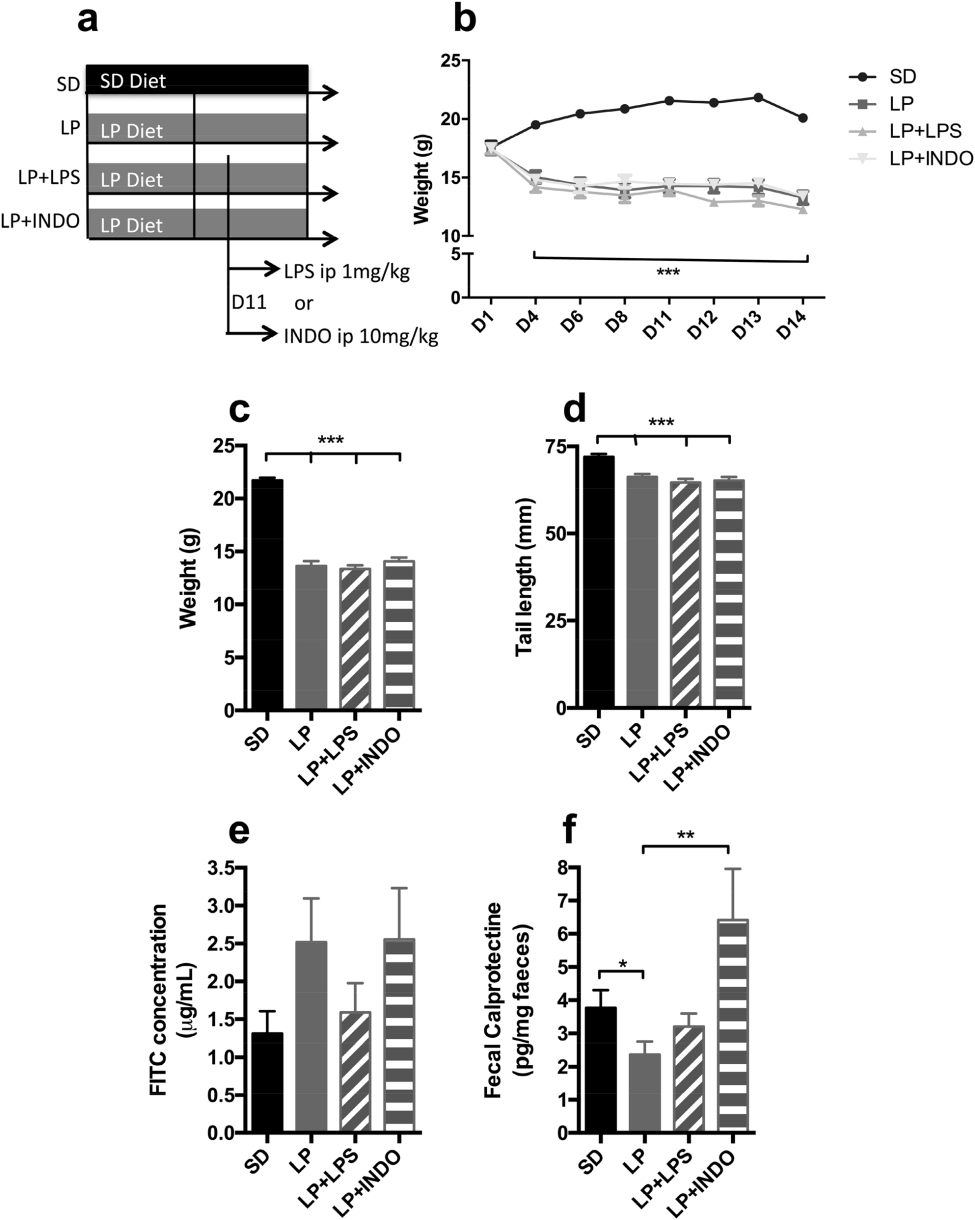
Growth, intestinal inflammation and permeability assessment in C57BL/6 mice fed a low protein in combination with lipopolysaccharide or indomethacin i.p. injection. (**a**) 3-weeks-old mice were fed with standard or isocaloric low protein diet post weaning during 14 days. At day 11, mice received a single intraperitoneal injection of LPS (1 mg/kg) or indomethacin (10 mg/kg). (**b**) Body weight was recorded at D1, D4, D6, D8 and every day from D11 until the end of the experimentation (n = 20 per group). Plots represent the mean ± SEM (***P < 0.001 vs. SD, Two Way ANOVA). At D14, (**c**) weight (n = 20 per group) and (**d**) tail length (n = 20 per group) were calculated. Bars indicate the mean ± SEM. One-way analysis of variance with post hoc Tukey’s test was performed (***P < 0.0001). (**e**) Bars indicate the mean ± SEM of the FITC concentration serum (assessed 3 h post *per os* administration; n = 20 per group). Kruskal Wallis test with post hoc Dunn's multiple comparison test was performed. (**f**) Bars indicate the concentration of fecal calprotectin in mice at D21 (n = 20 per group). Unpaired t-test (*p = 0.0423) or Mann–Whitney test was performed (**p = 0.0044). SD, Standard diet; LP, Low protein diet; LP + LPS, Low protein diet + i.p. injection of lipopolysaccharides; LP + INDO, LP + i.p. injection of indomethacin.

In order to induce gut barrier alteration, low-protein diet was associated to a gastrointestinal damage trigger and we investigated two approaches: liposaccharides (LPS) or indomethacin (Fig. 2a). All LP groups had a lower body weight (− 37.2% for LP, − 38.6% for LP + LPS, − 35.3% for LP + INDO, P < 0.0001 each vs. SD—Fig. 2b,c) and shorter tail length (− 8% for LP, − 10% for LP + LPS, − 9% for LP + INDO, P < 0.0001 each vs. SD—Fig. 2d). LP groups had lower mRNA levels for cytokines IL-1β, TNFα and MCP-1 (Supplementary Figure 1, P = 0.0152; P = 0.0184; P = 0.0246 respectively). In combination with low-protein diet, LPS and indomethacin administration did not worsen growth deficit (Fig. 2d). LPS administration did not affect intestinal permeability (+ 21.3% for LP + LPS, P = 0.8 vs. SD—Fig. 2e), fecal calprotectin levels (− 15% for LP + LPS, P = 0.4 vs. SD—Fig. 2f), mRNA levels for TNFα and MCP-1 (Supplementary Figure 1, P = 0.0749; P = 0.0522 vs. SD + LPS respectively) but significantly decreased mRNA levels for IL-1β (Supplementary Figure 1, P = 0.0117 vs. SD + LPS). Intraperitoneal injection of indomethacin did not alter significantly intestinal permeability (+ 94.7% for LP + INDO, P = 0.4 vs. SD—Fig. 2e) but increased fecal calprotectin levels compared to mice only fed with LP diet (+ 170% for LP + INDO, P = 0.0044 vs. LP—Fig. 2f).

### Effect of a single indomethacin gavage with 5-days LP diet on growth and barrier function (Fig. 3)

**Figure 3:**
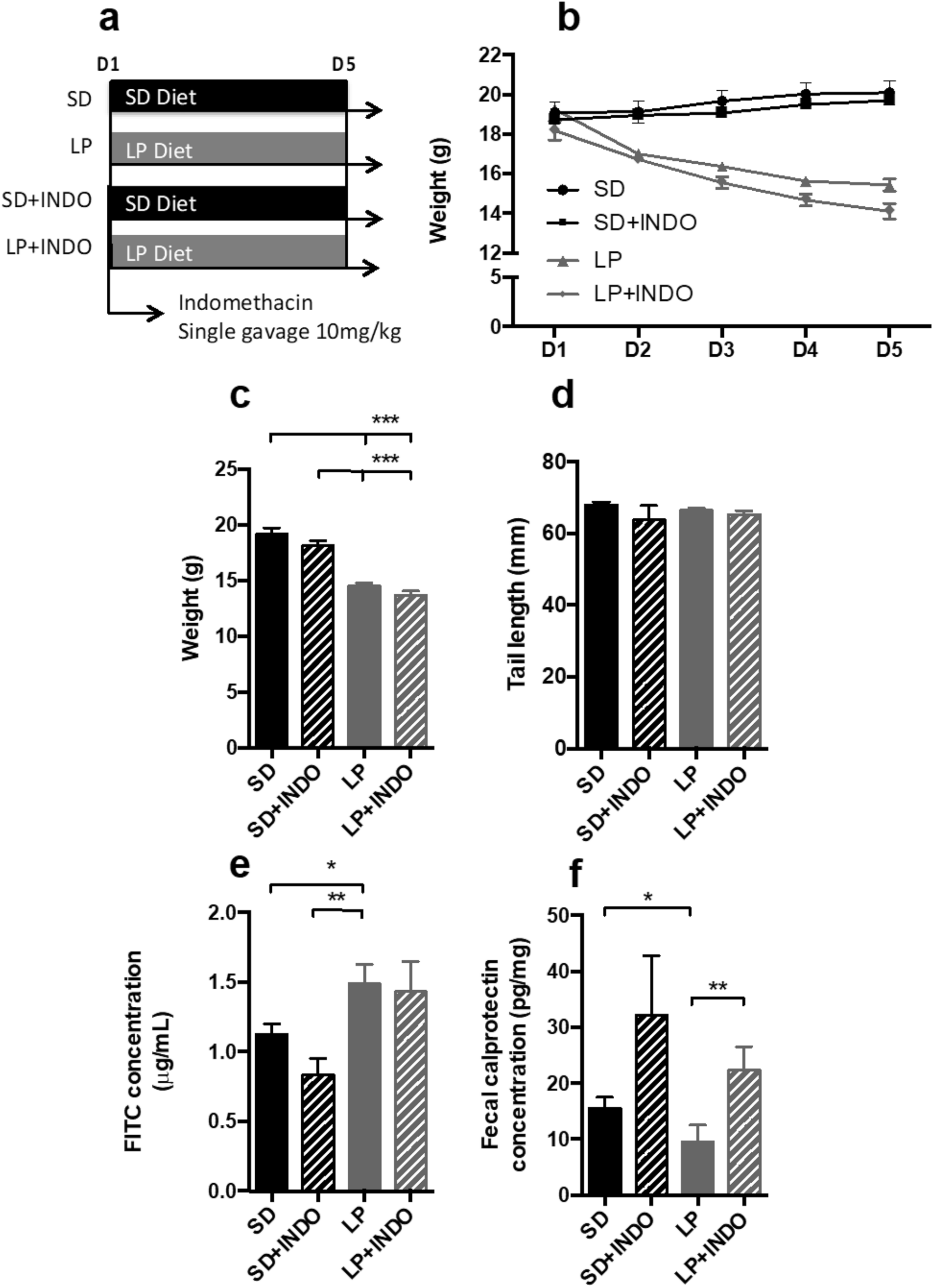
Growth, intestinal inflammation and permeability assessment in C57BL/6 mice fed a low protein combination with a single indomethacin gavage. (**a**) 3-weeks-old mice were fed with standard or low protein diet for 5 days. At day 1, a single gavage of indomethacin (10 mg/kg) was performed. (**b**) Body weight was recorded every day until the end of the experimentation (n = 20 per group). Plots represent the mean ± SEM (***P < 0.0001, Two Way ANOVA). At D5, (**c**) body weight (n = 20 per group) and (**d**) tail length (n = 20 per group) were measured. Bars indicate the mean ± SEM Kruskal–Wallis test with post hoc Dunn’s test was performed (***P < 0.0001). (**e**) Bars indicate the mean ± SEM of the serum FITC concentration (assessed 3 h post *per o*s administration; n = 20 per group). One-way analysis of variance with post hoc Tukey’s test (**P < 0.0041) and unpaired t-test (*p < 0.05) was performed. (**f**) Bars indicate the concentration of fecal calprotectin in mice at D21 (n = 20 per group). Mann–Whitney test was performed (**p = 0.0011; *p = 0.0037). SD, Standard diet; LP, Low protein diet; LP + INDO, LP + i.p. injection of indomethacin.

As indomethacin injection had an impact on intestinal inflammation, we thus speculated that oral gavage with indomethacin may have a stronger effect on intestinal permeability (Fig. 3a)^19^. Mice fed with low-protein diet for 5 days gained less weight than mice fed with SD (− 24% for LP, P < 0.0001 vs. SD—Fig. 3b,c). Five-day LP diet was not sufficient to impact linear growth (− 2.3% for LP, P = 0.1174 vs. SD) as shown by the absence of difference in tail length among groups (Fig. 3d). By contrast, 5-days-LP diet decreased fecal calprotectin (− 38% for LP, P = 0.0011 vs. SD—Fig. 3f) and induced intestinal hyperpermeability (+ 32% for LP, P = 0.03 vs. SD—Fig. 3e). No additional effect of oral indomethacin administration was observed on growth parameters or EED features (Fig. 3e,f).

### Effect of a 3-weeks LP diet combined with repeated indomethacin gavages on growth and barrier function (Figs. 4, 5 and 6)

**Figure 4:**
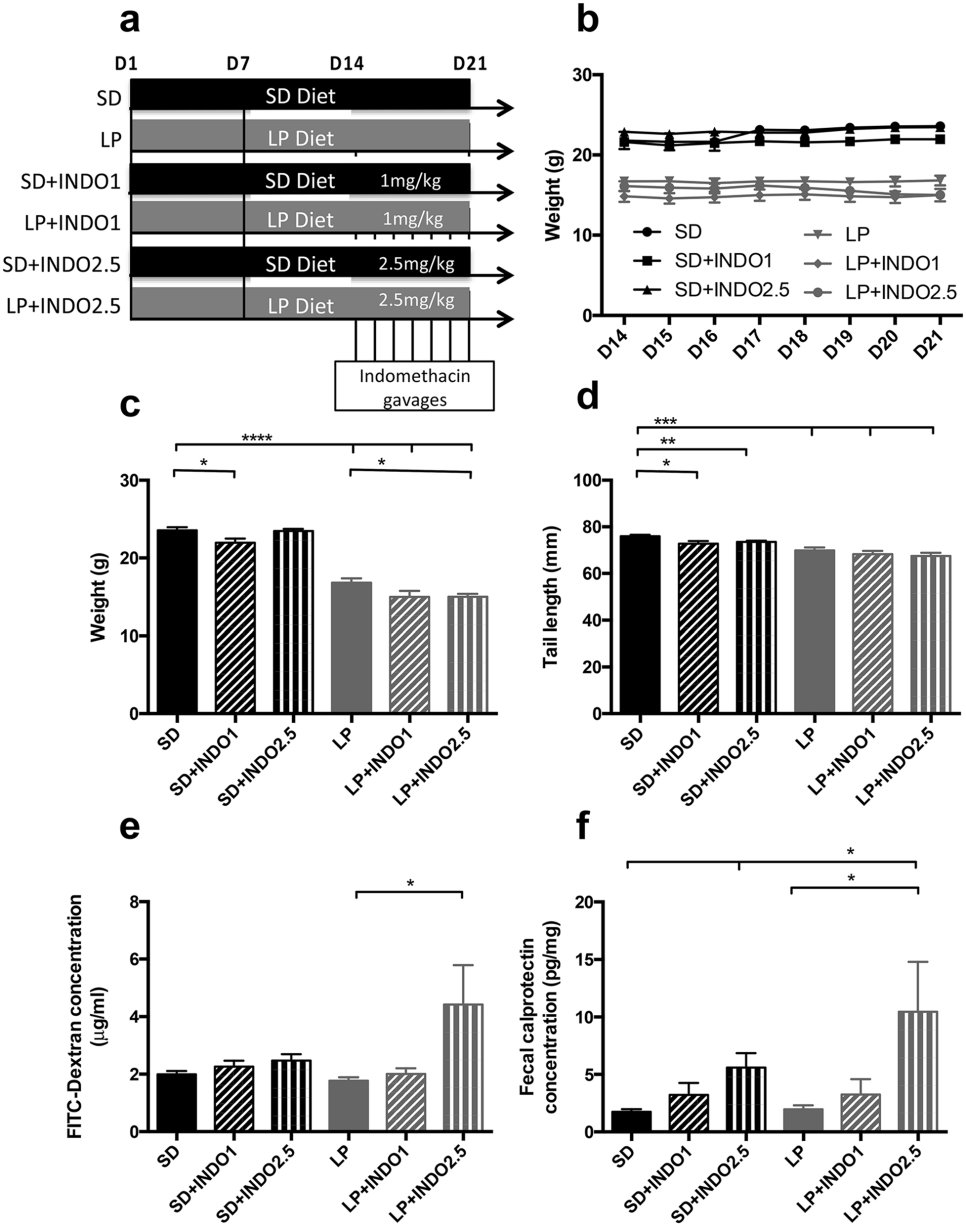
Growth, intestinal inflammation and permeability assessment in C57BL/6 mice fed a low protein or isocaloric standard diet combined with chronic indomethacin gavages. (**a**) 3-weeks-old mice were fed with standard or low protein diet for 21 days. At day 14, indomethacin (1 or 2.5 mg/kg) gavage was performed once a day for 7 days. (**b**) Body weight was recorded every day from D14 until the end of the experimentation (n = 10 per group). Plots represent the mean ± SEM (***P < 0.001, Two-way ANOVA). At D21, (**c**) body weight (n = 9–10 per group) and (**d**) tail length (n = 9–10 per group) were measured. Bars indicate the mean ± SEM One-way analysis of variance with post hoc Tukey’s test was performed (***P < 0.0001). (**e**) Bars indicate the mean ± SEM of the serum FITC concentration (assessed 3 h post *per os* administration; n = 7–10 per group). Mann–Whitney test was performed (*p = 0.0418). (**f**) Bars indicate the concentration of fecal calprotectin in mice at D21 (n = 7–10 per group). Unpaired t-test (*p = 0.0106) or Mann–Whitney (**p = 0.0012) test was performed. SD, Standard diet; SD INDO 1 Standard Diet + indomethacin gavages (1 mg/kg); SD INDO 2.5 Standard Diet + indomethacin gavages (2.5 mg/kg); LP, Low protein diet; LP INDO 1 LP + indomethacin gavages (1 mg/kg); LP INDO 2.5 LP + indomethacin gavages (2.5 mg/kg).

**Figure 5:**
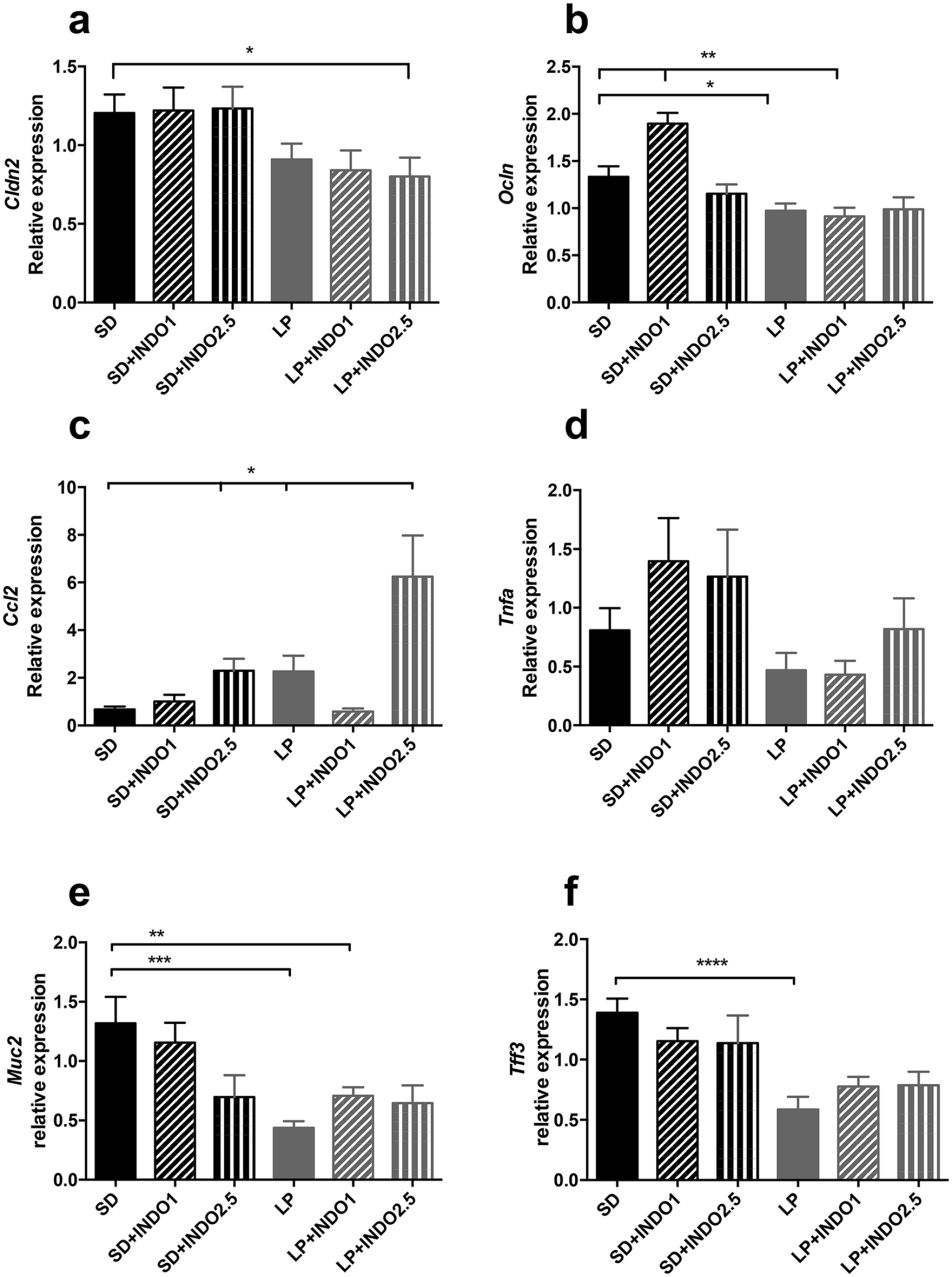
Cytokine and tight junction proteins RNA expression assessment in C57BL/6 mice fed a low protein or isocaloric standard diet combined with chronic indomethacin gavages Jejunal tight junction protein mRNA relative expression as (**a**) *Cldn2* (t-test *p = 0.0336); (**b**) *Ocln* (t-test **p < 0.01; *p = 0.0171) were measured. Proinflammatory cytokines mRNA levels in the jejunum as (**c**) *Ccl2* (t-test *p < 0.05) and (**d**) *Tnfa* were measured. Protective components of gut barrier (**e**) *Muc2* (t-test **p < 0.0132, ***p < 0.0013) and (**f**) *Tff3* (Mann–Whitney test ****p < 0.0005) mRNA relative expression were performed. Bars indicate the mean ± SEM Unpaired t-test or Mann–Whitney test was performed. SD, Standard diet; SD INDO 1 Standard Diet + indomethacin gavages (1 mg/kg); SD INDO 2.5 Standard Diet + indomethacin gavages (2.5 mg/kg); LP, Low protein diet; LP INDO 1 LP + indomethacin gavages (1 mg/kg); LP INDO 2.5 LP + indomethacin gavages (2.5 mg/kg).

**Figure 6:**
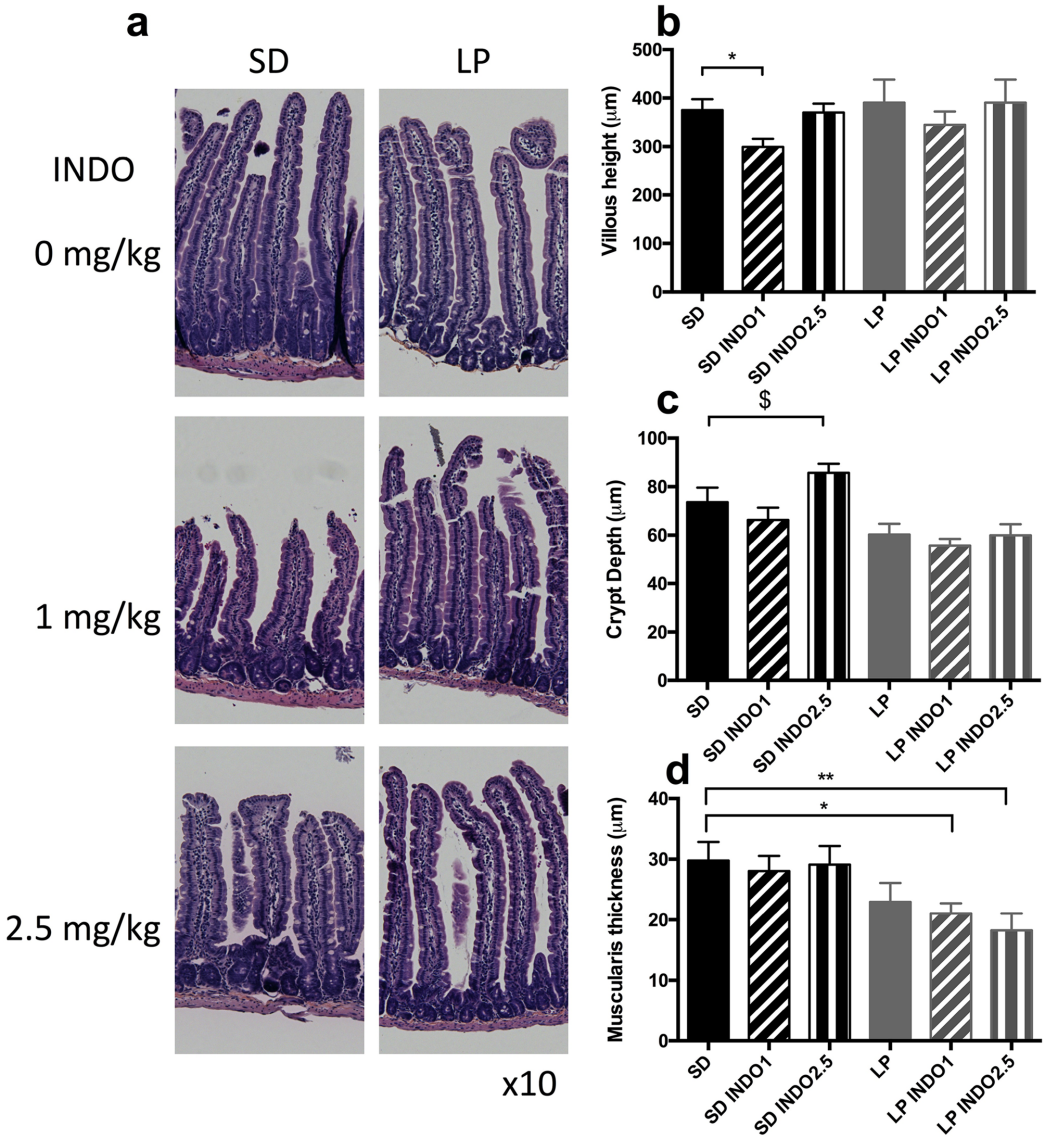
Villi length, crypt depth and muscularis thickness in C57BL/6 mice fed a low protein or isocaloric standard diet combined with chronic indomethacin gavages. (**a**) Jejunal section stained with HES solution were used to assess Intestinal damages. (**b**) Villi length (t test *P = 0.0186 vs. SD), (**c**) crypt depth (Mann–Whitney ^$^P = 0.0714 vs. SD) and (**d**) *muscularis* thickness (t test *P = 0.0254 vs. SD − Mann–Whitney **P = 0.0283 vs. SD) were measured. Unpaired t-test or Mann–Whitney test was performed SD, Standard diet; SD INDO 1 Standard Diet + indomethacin gavages (1 mg/kg); SD INDO 2.5 Standard Diet + indomethacin gavages (2.5 mg/kg); LP, Low protein diet; LP INDO 1 LP + indomethacin gavages (1 mg/kg); LP INDO 2.5 LP + indomethacin gavages (2.5 mg/kg).

Daily oral indomethacin at 1 or 2.5 mg/kg (Fig. 4a) did not induce mice mortality. LP diet or oral indomethacin treatment had a significative impact on body weight or tail length (2-way ANOVA, no interaction, dietary effect, indomethacin effect, all P < 0.05) (Fig. 4b–d). The combination of LP diet with indomethacin had also a significative impact on EED features: increased intestinal permeability (+ 150% for LP + INDO2.5, P = 0.0418 vs. LP—Fig. 4e) and gut inflammation assessed by fecal calprotectin (+ 496% for LP + INDO2.5, P = 0.0012 vs. SD) (Fig. 4f). The combination of 3 weeks-LP diet with 1 week daily oral gavage of indomethacin significantly decreased *Cldn2* (Claudin-2) by 33% (P = 0.0336 vs. SD—Fig. 5a) and decreased *Ocln* (Occludin) mRNA levels by 26% (P = 0.0587 vs. SD—Fig. 5b). The combination of LP diet with oral indomethacin at 2.5 mg/kg significantly increased *Ccl2* (Monocyte-chemoattractant protein 1/MCP-1) mRNA level (+ 829% for LP + INDO2.5, P = 0.02 vs. SD) compared to mice fed with SD diet (Fig. 5c) without impact on *Tnfa* (TNFα) mRNA levels (Fig. 5d). LP diet decreased jejunal *Il1b* (Interleukin 1mRNA level (− 47% for LP p = 0.0132 vs. SD) without additional impact of indomethacin gavages (data not shown). LP diet alone significantly decreased jejunal *Muc2* (Mucin-2) (− 33% for LP, p < 0.001 vs. SD Bonferroni post-test— − 53% for LP + INDO1, p < 0.01 vs. SD Bonferroni post-test) and *Tff3* (Trefoil Factor 3) (− 42% for LP, p < 0.01 vs. SD Bonferroni post-test) mRNA levels (2-way ANOVA, interaction p < 0.05, diet effect P < 0.05, no indomethacin effect) (Fig. 5e,f).

Indomethacin gavages (1 mg/kg) induced villus blunting (− 20% for SD + INDO1, P = 0.0186 vs. SD—Fig. 6a,b). No significative impact of LP diet or LP diet combined with indomethacin gavage in villi length or crypt depth was detected (Fig. 6b,c). LP diet and indomethacin combination reduced *muscularis* thickness (− 29% for LP + INDO1, P = 0.0254 vs. SD; − 39% for LP + INDO2.5; P = 0.0283 vs. SD—Fig. 6d) while LP alone did not.

## Discussion

In the present study, we developed a murine model combining undernutrition with enteropathy resulting in (i) wasting and stunting, (ii) inflammation and (iii) gut hyperpermeability.

We first investigated the impact of undernutrition alone for 3 weeks by limiting the amount of calories by 50% or by feeding mice with a LP diet. Both approaches had a significant impact on growth by reducing body weight and exhibiting shorter tails. These approaches had no effect on gut barrier function or intestinal permeability. Caloric restriction-induced undernutrition models are used in the literature from 15 to 50% of caloric restriction but are not associated with gut barrier dysfunction^8^. Dietary protein restriction is used from 0 to 7% of proteins and are associated with gut barrier dysfunction, only in the case of drastic dietary protein restriction^8^. Similarly, rats fed with LP diet (4% protein) for 20 days had growth retardation without effect on colonic or ileum permeability to macromolecules^10^. Brown et al. study showed no impact of 3 weeks of LP diet (7%) in weaning mice on fecal calprotectin levels^9^ while it increased intestinal permeability^9^. By exposing mice to protein malnutrition from 5 or 14 days, fecal calprotectin levels decreased while a longer exposure for 21 days had no influence on these levels. We speculated that it may result from physiological adaptations. First, mice are able to cope with protein malnutrition and they develop survival-promoting strategies with a reduced inflammatory state. Then, there is a progressive exhaustion of the adaptive mechanisms. A similar pattern was observed in malnourished mice with focal cerebra ischemia^21^. While malnutrition for 7 to 14 days induced survival-promoting mechanisms such as a neuroprotection and immunoregulation, longer exposure to malnutrition for 30 days impairs stroke outcome^21^.

As dietary protein deficiency had a higher effect on body weight compared to caloric restriction and a lower impact on animal’s welfare^22^, we further chose low protein diet to reproduces undernutrition features. As dietary protein deficiency alone was not sufficient to impact gut barrier, we then investigated the combination of LP diet with a compound triggering a gut barrier insult. We thus explored two approaches: bacterial LPS and indomethacin. Bacterial LPS is the main cell wall component of gram-negative bacteria and increased anti-LPS Immunoglobulins concentrations in children are associated to poor growth outcomes^16,17^. In our experimental design, intraperitoneal injection of LPS in combination with LP diet, did not result in poorer growth than LP diet alone and did not induce EED features. LPS injection at the same dose was able to induce intestinal permeability in normo-nourished mice^23^. We thus speculated that undernutrition may impair the inflammatory response to a LPS challenge. Indeed, we observed that LP diet for 2 weeks decreased mRNA levels for cytokines. In addition, Neyestani et al*.* showed altered immunity response after 14 days with LP diet (2%) in weaning mice^24^. This mechanism has already been described in central inflammation^25^. Actually, LP diet during pregnancy decreased offspring inflammatory response to acute LPS in the hypothalamus^25^. Indomethacin, a non-steroidal anti-inflammatory drug has been used to induce enteropathy in experimental models^19,20^. Indomethacin challenge at 10 mg/kg in malnourished mice did not worsen body weight loss or growth faltering but increased fecal calprotectin levels compared to mice fed with LP diet. Similar results on intestinal inflammation were found in non-malnourished mice from day 1 to day 4 after a single injection of indomethacin at the same dose^26,27^. No impact of indomethacin at 10 mg/kg in gut permeability was observed in malnourished mice in the present study.

To increase the effect of indomethacin on intestinal barrier, we orally exposed mice to a single gavage of indomethacin before LP diet started and studied the impact 1 week later. No additional effects of oral indomethacin administration compared to LP diet alone were observed on growth parameters or EED features. Concerning intestinal permeability, Jacob et al. observed higher intestinal permeability 1–6 h in rats receiving indomethacin gavage at 20 mg/kg and returned to normal 4 days post treatment^28^. We thus hypothesised that indomethacin-induced increase in intestinal permeability may be transient.

To strengthen indomethacin impact on gut barrier, we then set up repeated gavages of indomethacin. Indeed, Whitfield-Cargile et al. demonstrated that 6 days of repeated indomethacin gavage at 5 mg/kg led to enteropathy in well-nourished mice^20^. Post-weaning mice were therefore fed with LP diet or SD diet for 3 weeks. In our murine model, much of the body weight loss and the lower tail length was driven by the low protein diet as expected^9^ however, indomethacin exposure also induced a lower body weight. Body weight loss is observed in indomethacin-treated mice^29^. It may result from multiple mechanisms such as (i) intestinal damage leading to a reduced food intake, (ii) decreased cyclooxygenase-2 expression leading to decreased mucosal protection^19^, (iii) microbiota changes^29^. Xiao et al. have shown that microbiota depletion by antibiotics treatment improved body weight in indomethacin-treated mice^29^. Neither dietary exposure, nor indomethacin treatment alone were able to induce intestinal permeability but the combination of both induced intestinal hyperpermeability. Low protein diet is not sufficient to impact intestinal permeability but can alter gut barrier components such as mRNA levels for *Muc2, Tff3 and Ocln*. It may create a favourable environment to increase susceptibility to the negative effects of indomethacin. It has been demonstrated that infection by worms is more severe in mice fed with low protein diet^30^. In addition, a recent study demonstrated that MUC2 deficient mice are more susceptible to sepsis^31^.

This discrepancy between dietary impact on intestinal permeability and gut barrier components is already documented in the literature^32^ while these terms are often used interchangeably. We have previously shown that dietary supplementation by l-Glutamine was able to restore intestinal permeability without changing occludin mRNA levels in mice with activity-based anorexia^33^. We hypothesized that this vulnerability may be the result of microbiota changes. Indeed, Brown et al. have shown that low protein diet induced an altered small intestinal microbiota^9^ and Xiao et al. have demonstrated the role of microbiota changes in response to indomethacin treatment^29^. The combination of LP diet with indomethacin also had a significant impact on gut inflammation assessed by fecal calprotectin. This is in accordance with Whitfield-Cargile et al. showing that chronic indomethacin gavage at 5 mg/kg for 6 days increased fecal calprotectin^20^.

In the present study, the combination of 3 weeks LP diet with chronic oral gavage of indomethacin significantly decreased *Cldn2* and *Ocln* mRNA levels. These results are in accordance previous studies showing a reduced ileal and colonic occludin protein expression in protein-deprived rats^10,11^.

To better understand gut barrier response, we also studied *Muc2* and *Tff3* which contribute to epithelial protection. We observed that LP diet reduced jejunal *Muc2* and *Tff3* mRNA levels, suggesting a decrease in epithelium protection. Similarly, dietary protein restriction in pregnant dams alters barrier function by reducing *Muc2* and *Tff3* mRNA levels in colonic mucosa of offspring suggesting direct impact of undernutrition in mucus alteration^34^. By contrast, dietary high protein diet increased ileal *Muc2* mRNA expression in rats^35^. The combination of LP diet with oral indomethacin at 2.5 mg/kg increased jejunal *Ccl2* mRNA level compared to mice fed with SD diet as previously described by Harusato et al*.* in C57BL/6 normonourished mice^36^. In contrast, *Il1b* and *Tnfa* mRNA remained unchanged. In our study, LP diet did not impact villous height or crypt depth and similar results were observed after 26-days fed with LP diet (2%) or 3-weeks fed with LP diet (7%)^9,37^. In contrast, repeated indomethacin gavages induced villous blunting as previously shown^20,38^.

Throughout our four experiments, we observed that mice body weight decreased by exposing mice to protein malnutrition from 5, 14 or days, while dietary exposure to a shorter protein malnutrition for 5 days is not sufficient to impact tail length. Similarly, a longer protein malnutrition exposure is necessary to induce a vulnerable environment to enable indomethacin impact on gut barrier function.

Our present murine model reflects features observed in children with undernutrition and EED. LP diet reflects the poor nutritional environment leading to undernutrition. LP diet was able to induce wasting and linear growth failure but was not sufficient alone to impact gut barrier function. By repeated oral exposure to indomethacin, a gut barrier dysfunction was induced. Higher intestinal permeability was observed as described in human EED^39^ and this effect was confirmed by decreased mRNA levels encoding for tight junction proteins. Fecal calprotectin, a proposed biomarker in EED^40^, was also increased in the present model. Human EED is also characterized by inflammatory cell infiltrate^41^ and we detected increased jejunal *Ccl2* mRNA levels for MCP-1 suggesting this infiltration also occurred in our model.

As higher endotoxins levels have been observed in EED^15^, we initially speculated that LPS would be more relevant to use than indomethacin in order to induce EED. Through our experiments, we finally demonstrated the opposite. Indomethacin use may not reproduce identical mechanisms involved in EED etiology but enabled to display many EED features while LPS did not. Experimental models of enteropathy induced by chemical agents such as indomethacin are used in the analysis of pathological mechanisms of enteropathy^29,42^ as well as the development of therapeutic agents^43^. Although NSAID-induced enteropathy models do not have the complexity of human EED, the present model can contribute to the study of the disease such as microbiota changes^29^ or to the evaluation of nutritional intervention^44^. In addition, use of indomethacin provides an easy and reproducible model with a more controlled inflammatory response that may compare to subclinical symptoms found in human pathophysiology. The present model recapitulates key features of human EED such as growth failure, intestinal hyperpermeability and inflammation and is comparable to the model developed by Brown et al. using LP diet and bacterial challenge^9^. Brown’s model with bacterial challenge reflects more a primary mechanism of the human disease but our model encompasses methodologies that are considered easy to induce, and its simplicity allows it to be used in several experimental protocols. While regional LP diets such as Regional Basic Diet from Northeast Brazil consider geographic differences in EED development, we chose a commercial LP diet as used in Brown’s model.

In addition, human EED is a complex syndrome with multiple phenotypes depending on various adverse exposure^45^ and geographic differences^46^ and no single model captures the complexity of human EED^8^. Nevertheless, the present model provides valuable insights into key EED features to better understand mechanisms behind the human disease.

**Figure 7:**
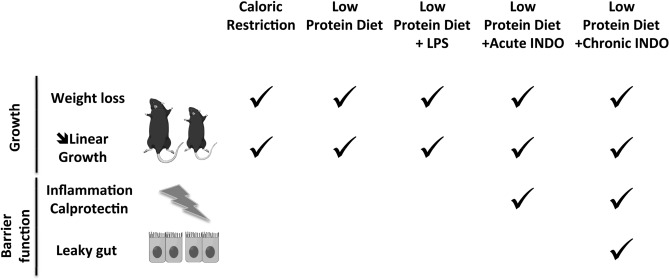
Undernutrition leads to growth failure and is often associated to environmental enteric dysfunction (EED). We aimed to develop a preclinical model of undernourished model with environmental enteropathy features such high intestinal inflammation and intestinal permeability. To induce undernutrition with EED, post-weaning C57BL/6 mice were fed with malnourished diet alone or combined with a gastrointestinal insult trigger. Growth was assessed by body weight and tail length. Intestinal permeability and inflammation were used as enteropathic markers. CR and LP for 3 weeks induced stunting and wasting but had no intestinal impact. We therefore decided to combine LP diet to a gastrointestinal insult trigger by liposaccharides (LPS) or indomethacin. LPS did not significantly impact small intestine while indomethacin increased fecal calprotectin production. To accentuate the effects, we investigated the effects of repeated gavages of indomethacin in addition to LP diet and mice exhibited stunting and wasting with intestinal hyperpermeability and gut inflammation.

In conclusion, we developed a murine model of undernutrition with EED features (intestinal inflammation and hyperpermeability) that may be compared to what may be observed in humans (Fig. 7), and particularly in children aged less than 5 years who become severely wasted prior to or after having accumulated significant linear growth retardation. Understanding the pathophysiological mechanisms involved during an episode of undernutrition (wasting and /or stunting) associated to EED is a critical step to develop novel therapeutic strategies. Obvious methodological limitations hamper the investigation of gut function in undernourished children, and particularly the lack of validated non-invasive methods. A stable and reproducible animal model is therefore an interesting and affordable tool to elucidate pathophysiological processes and potentially evaluate innovative therapeutic applications.

## Methods

### Ethics

Animal care and experimentation complied according to the European directive for the use and care of laboratory animals (2010/63/UE) and received the agreement of the local animal ethics committee (Comité National de Réflexion Ethique sur l'EXpérimentation Animale) and of the ministerial committee for animal experimentation (registration number: APAFIS#6185). Animal welfare was monitored daily by visual inspection. All interventions were done during the light cycle and mice were given paper nesting material as enrichment.

### Animals and treatment regimen

The murine model was initiated at post-weaning stage to enable further studies investigating the impact of early-life nutritional interventions to limit or reverse EED development. Post weaning 3-week-old male mice C57BL/6 were ordered for each experiment (Janvier, Le Genest-Saint-Isle, France). They were housed to a cage and acclimatized for 1 week. During this period, they received standard diet ad libitum (A03 21.4% protein, 5.1% fat—SAFE, Augy, France) and had access to tap water. All experiments took place in a climate-controlled facility with 12/12 light/dark cycle and mice were randomized per cage and assigned to a specific regimen. At the end of each protocol, mice were killed by the intraperitoneal administration of a combination of lethal anesthetics (ketamine 40 mg/kg plus xylazine 1 mg/kg). Samples were stored immediately at − 80 °C.

#### Experiment 1—effect of protein or caloric restriction

Mice were fed with either standard (SD, 21.4% protein, 5.1% fat, n = 20—SAFE A003), isocaloric low protein (LP, 5.8% protein, 6% fat, n = 20—SAFE) diet ad libitum or standard diet with 50% caloric restriction (CR) for 21 days (Table 1, Fig. 1a). CR were calculated from CT mice pellet consumption (3.5 g/day) and divided by 2 (1.75 g/day).

**Table 1:** Standard; caloric restriction and low protein diet composition and consumption.

	Control diet (SD)		− 50% caloric restriction		Low protein diet (LP)	
	g%	kcal%	g%	kcal%	g%	kcal%
Protein	21.4	27.3	21.4	27.3	5.8	6.9
Carbohydrates	52.0	66.2	52.0	66.2	72	85.9
Fat	5.1	6.5	5.1	6.5	6	7.2
Total		100		100		100
kcal/g	3.395		3.395		3.395	
Mice						
Consumption/day (g)	3.5		1.75		3.5	

	Control diet (SD)		− 50% caloric restriction		Low protein diet (LP)	
	g		g		g	
Protein	0.749		0.3745		0.203	
Carbohydrates	1.82		0.91		2.520	
Fat	0.1785		0.08925		0.210	
Total energy/day (kcal)	12		6		12	

#### Experiment 2—impact of undernutrition and liposaccharides or indomethacin single intraperitoneal injection

Mice were fed with either standard (SD, n = 20) or isocaloric low protein diet for 14 days. At D11, mice received a single intraperitoneal injection of either lipopolysaccharide (1 mg/kg, LP + LPS, n = 20-Serotype O111:B4, Sigma Aldrich), indomethacin (10 mg/kg, LP + INDO, n = 20, Sigma Aldrich) diluted in water or vehicle (LP, n = 20, Fig. 2a).

#### Experiment 3—impact of undernutrition and single gavage of indomethacin

Mice were fed with either standard or isocaloric low protein diet for 5 days. At D1, a single gavage of indomethacin (10 mg/kg, n = 20) diluted in 1% carboxymethylcellulose (Sigma Aldrich) was performed while control groups were gavaged with vehicle (Fig. 3a).

#### Experiment 4—impact of undernutrition and repeated gavages of indomethacin

Mice were fed with either standard or isocaloric low protein diet for 21 days. At D14, indomethacin (1 or 2.5 mg/kg, n = 10 for each group) diluted in dimethyl sulfoxide^20^ was given while control groups were gavaged with vehicle. Gavages were performed once a day for 7 days (Fig. 4a).

### Growth and body weight

Body weight was recorded every 2 days and tail length was measured to assess linear growth at the end of the protocol.

### Fecal calprotectin concentration

Mice feces were collected at the end of each experiment, weighted, homogenized in 500 µL PBS + 1% inhibitors (protease and phosphatase inhibitor cocktail, Sigma Aldrich) and centrifuged at 13,000*g*, 20 min. Supernatant was stored at − 80 °C. Fecal calprotectin measurement was performed on supernatants using a S100A8/S100A calprotectin ELISA kit following manufacturer’s instructions (R&D System, Mineapolis, USA). Concentration was determined by assessing the OD 450 nm using a plate reader (Tecan, Männedorf, Suisse), comparing with a standard curve of known concentration of calprotectin.

### Intestinal permeability assessment

Jejunal permeability was assessed by measuring 4 kDa Fluorescein-isothiocyanate (FITC, Sigma Aldrich)-dextran mucosal to serosal flux level in Ussing chambers (Harvard Apparatus, Holliston, United States) as previously described^47^. Intestinal permeability was assessed by serum FITC-dextran flux concentration^9^. Briefly, mice were fasted 6 h before FITC-dextran gavage (60 mg/kg). Three hours post gavage, plasma was collected *post-mortem*. FITC-dextran fluorescence level was measured by using a 96-well black plate reader (Chameleon V—Hidex, Turku, Finland) read with the excitation of 485 nm and emission of 530 nm. A standard curve was used to convert values to concentration.

### RT-q-PCR

First, 1.5 μg total RNA into cDNA by using 200 units of SuperScript II Reverse Transcriptase (ThermoFischer, Whaltham, Massachussets, USA) was used for reverse transcription as previously described^48^. SYBR Green technology on BioRad CFX96 real time PCR system (BioRad Laboratories, Marnes la Coquette, France) was used to perform qPCR in duplicate for each jejunal sample as previously described^48^. *Gapdh* (glyceraldehyde-3-phosphate dehydrogenase), *B2m* (Beta-2-Microglobulin) and *Rn18s* (18S ribosomal RNA) were used as reference genes. Sense and anti-sense primers are described in the supplementary Table 1.

### Histology

Jejunum samples from experiment 4 were embedded in paraffin. Sections of 4 mm were cut with a microtome and stained with a solution of hematoxylin–eosin-saffron (HES) as previously described^49^. Samples were blinded and analyzed with photonic VisionTek Live Digital Microscope (Sakura, The Netherland). Histological analysis of jejunal villi height (μm), crypt depth (μm) and jejunal *muscularis* thickness (μm) were obtained with VisionTeck software (Sakura).

### Statistics

Results were expressed as mean ± standard error mean (SEM) and were compared using GraphPad Prism 5.0 (GraphPad Software, La Jolla, United States). Inter-individual comparisons between two groups were performed with parametric Student t test or non-parametric Mann–Whitney test, and ANOVA followed by Tukey post-tests for more than two groups. P value < 0.05 was considered significant. Indomethacin and LP diet effects were analyzed by two-way analysis of variance for repeated measures with Tukey’s post hoc tests.

## Supplementary information

Supplementary Information 1.

Supplementary Information 2.

## Abbreviations

*B2m*: β-2-Microglobulin
*Cldn2*: Claudin-2
*Ccl2*: Monocyte-chemoattractant protein 1
CR: Caloric restriction
EED: Environmental enteric dysfunction
FITC: Fluorescein-isothiocyanate
*Gapdh*: Glyceraldehyde-3-phosphate dehydrogenase
HES: Hematoxylin–eosin–saffron
*Il1b*: Interleukin 1β
INDO: Indomethacin
LP: Low protein
LPS: Lipopolysaccharides
*Muc2*: Mucin2
*Ocln*: Occludin
*Rn18s*: 18S ribosomal RNA
SD: Standard diet
*Tff3*: Trefoil factor family 3
*Tnfa*: Tumor necrosis factor alpha
