# Supplementary material for: Modeling undernutrition with enteropathy in mice
Source: Sci Rep. 2020 Sep 24;10:15581. doi: 10.1038/s41598-020-72705-0 (PMC7518247; doi:10.1038/s41598-020-72705-0)

Modeling undernutrition with enteropathy in mice

Emmeline SALAMEH, Marine Jarbeau, Fanny B. Morel, Mamane Zeilani, Moutaz Aziz, Pierre Déchelotte, Rachel Marion-Letellier

Supplementary Figure 1: mRNA levels for pro-inflammatory cytokines in C57BL/6 mice fed a low protein in combination with lipopolysaccharide or indomethacin i.p. injection. 3-weeks-old mice were fed with standard or isocaloric low protein diet post weaning during 14 days. At day 11, mice received a single intraperitoneal injection of LPS (1mg/kg). Proinflammatory cytokines mRNA levels in the jejunum as (a) *Il-1beta,* (b) *Tnfa* and (c) *Mcp-1* were measured. SD, Standard diet; LP, Low protein diet; LP+LPS, Low protein diet + i.p. injection of lipopolysaccharides.

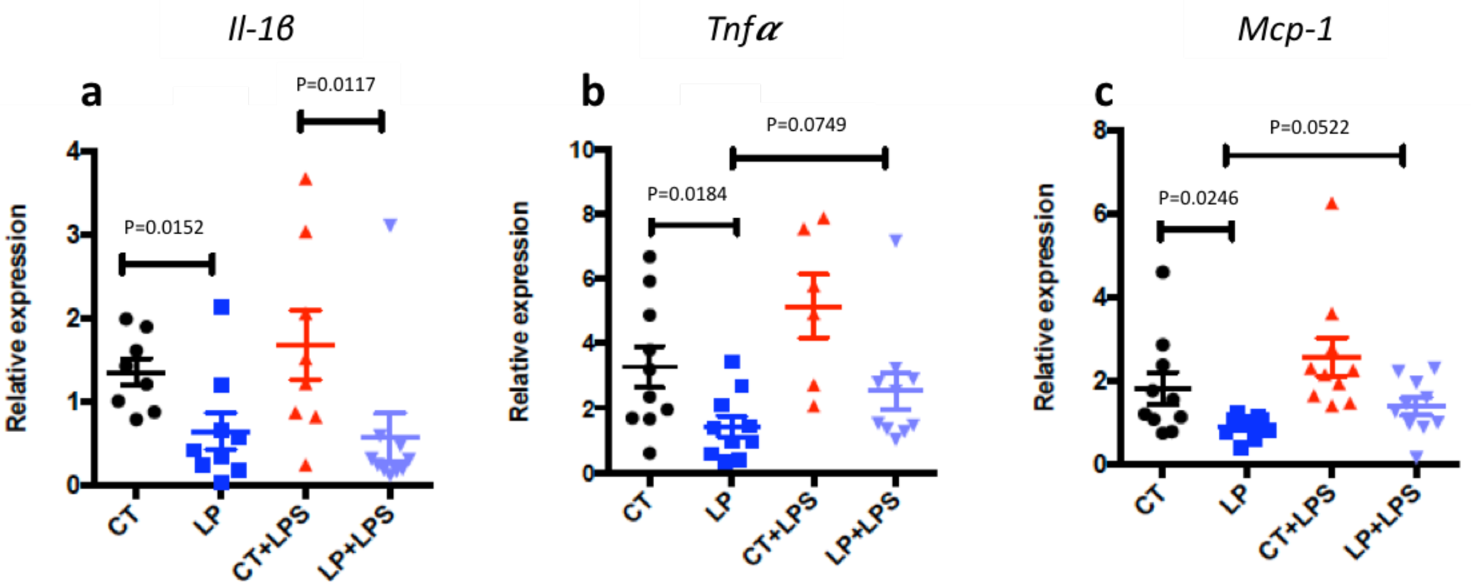

Supplement: Supplementary file 1 — Supplementary Information 1. [file 41598_2020_72705_MOESM1_ESM.pdf]
