# Supplementary material for: Modeling undernutrition with enteropathy in mice
Source: Sci Rep. 2020 Sep 24;10:15581. doi: 10.1038/s41598-020-72705-0 (PMC7518247; doi:10.1038/s41598-020-72705-0)
Supplement: Supplementary file 2 — Supplementary Information 2. [file 41598_2020_72705_MOESM2_ESM.pdf]

Modeling undernutrition with enteropathy in mice

Emmeline SALAMEH, Marine Jarbeau, Fanny B. Morel, Mamane Zeilani, Moutaz Aziz, Pierre Déchelotte, Rachel Marion-Letellier

Supplementary table 1: primers sequences

Name	Sequences
Rn18S	F: TGCGAGTACTCAACACCAACA<br>R: TTCCTCAACACCACATGAGC
B2m	F: GCCGAACATACTGAACTGCTAC<br>R: GCTGAAGAACATATCTGACATCTC
Cldn2	F: ATACTACCCTTTAGCCCTGACCGAGA<br>R: CAGTAGGAGCACACATAACAGCTACCAC
Gapdh	F: CATCACTGCCACTCAGAAGA<br>R: AAGTCACAGGAGACAACCT
Il1b	F: CCCAAAAGATGAAGGGCTGC<br>R: AAGGTCCACGGGAAAGACAC
Ccl2	F: TTAAAAACCTGGATCGGAACCAA<br>R: GCATTAGCTTCAGATTTACGGGT
Muc2	F: CGACACCAGGGATTTCGCTTAAT<br>R: CACTTCCACCCTCCCGGCAAAC
Ocln	F: CAGTAGGAGCACACATAACAGCTACCAC<br>R: CTGCAGACCTGCATCAAAAT
Tff3	F: CCTGGTTGCTGGGTCCTCTGG<br>R: GTCTCCTGCAGAGGTTTGAAGC
Tnfa	F: TGTCTACTCCTCAGAGCCCC<br>R: TGAGTCCTTGATGGTGGTGC
